# To Parents, Teachers, and Youth

**Published:** 1862-04

**Authors:** 


### HALL'S JOURNAL OF HEALTH.

Our Legitimate Scope is almost boundless: for whatever begets pleasurable and harmless feelings, promotes Health; and whatever induces disagreeable sensations, engenders Disease.

WE AIM TO SHOW HOW DISEASE MAY BE AVOIDED, AND THAT IT IS BEST, WHEN SICKNESS COMES, TO TAKE NO MEDICINE WITHOUT CONSULTING A PHYSICIAN.

Vol. IX.] APRIL, 1862. [No. 4.

### TO PARENTS, TEACHERS, AND YOUTH.

"I HAD rather see him in his grave," is the instinctive exclamation of a parent, in contemplating the choice for a child between death and a life-long confinement in a lunatic asylum; and yet, according to the uniform testimony of educated medical men at home and abroad, whose office it is to superintend those establishments founded for the restoration of the insane, thousands of persons languish every year in those institutions, and finally die in drivelling idiocy in consequence of practices fallen into unwittingly, and eventually habits formed in early youth, without the slightest idea of their being immoral or physically destructive. There are parents born, bred, and brought up in a pure, moral atmosphere, who are themselves profoundly ignorant of the existence of the things in question, and who, on the perusal of the following pages, will wisely and thankfully wake up, as if to the discovery of an unsuspected danger. The article is taken bodily from the Editor's book on "SLEEP," already within the year passed to a second edition.

